# The Salaries of Poor-Law Medical Officers

**Published:** 1914-04-04

**Authors:** 


### THE SALARIES OF POOR-LAW MEDICAL OFFICERS.

We have pointed out on several occasions recently that the difficulty of obtaining Poór-Law medical officers, especially for indoor posts, has compelled Boards of Guardians to offer increased salaries. Reports from all parts of the country show that the matter is now occupying the attention of many Boards of Guardians. In Holborn the Guardians, having had no applications for the post of second assistant medical officer at their infirmary when offered at a salary of £110 per annum, have decided to increase it to £140. At Droitwich an advertisement for a district medical officer at £90 brought no reply, and the Guardians are now considering a letter from the local medical men, pointing out the inadequacy of the salary under present-day conditions, and have decided to offer increased terms. At Swansea one of the labour Guardians stigmatised the offer of £255 per annum for a resident workhouse medical officer as scandalously inadequate, and the matter has been referred back for further consideration. As a contrast to these, the Mutford and Lothingland Guardians have rejected by nineteen votes to sixteen a recommendation that the salary of their workhouse medical officer should be increased from £60 to £80.

The position in this Union is worthy of some further consideration. The workhouse has 257 inmates, and 121 of these are under medical treatment. Under his contract the medical officer has to supply all drugs. During the discussion several Guardians complained that the medical officer did not spend as much time as he ought to in the workhouse. To this accusation the medical officer replied that he gave as much time as he could afford for the salary he was paid. In this reply we reach the fundamental objection to inadequate salaries. They effect no saving, but lead to unnecessary expense, and, when the medical service is starved, to avoidable suffering. Business men know that success in the conduct of any undertaking depends upon paying the full market value to those in control, and that the best and most efficient is, in the long run, the cheapest. Unfortunately, the incapacity of Poor-Law Guardians is visited not upon themselves, but upon the sick and the ratepayers.

It is much to be regretted that the Local Government Board, in its capacity as guardian of the Metropolitan Common Poor Fund, so often uses its powers to place obstacles in the way of progressive Boards of Guardians who realise that it is their duty to provide the best medical skill available for the relief of the sick under their charge. We had occasion to refer to this in commenting upon the Board's refusal to sanction in full the proposal of the Camberwell Guardians to increase the salary of their medical superintendent. Two other instances have occurred since then. In Bermondsey the Guardians submitted a well-thought-out scheme for the reorganisation of their medical staff. The Local Government Board, whilst assenting to the scheme in general, suggested that the number of medical officers and the salaries allotted to them should be reduced. Fortunately, the Bermondsey Guardians stuck to their guns and defended their position in an argument which merits quotation in full. "The sick poor of the parish," they wrote, "are deserving of as much consideration in the matter of the best qualified medical and surgical talent as the hospital patients. For economical reasons it is advantageous to give the sick patients the best medical, surgical, and infirmary treatment rather than offer them an inferior treatment at apparently less cost for the moment, though in the end really of a more expensive nature."

Again, quite recently, in Shoreditch, the Guardians wished to assign to the post of first assistant medical officer at the infirmary the salary of £200, rising to £250, but the Local Government Board would assent to a maximum of £220 only. To those who know the position in the London infirmaries, and the urgent need there is to attract to them the best qualified medical men, it is almost inconceivable that the very Board which ought to foster every attempt to effect this object, should strive rather to retard progress by its ill-timed parsimony.

